# Startling Sweet Temptations: Hedonic Chocolate Deprivation Modulates Experience, Eating Behavior, and Eyeblink Startle

**DOI:** 10.1371/journal.pone.0085679

**Published:** 2014-01-09

**Authors:** Jens Blechert, Eva Naumann, Julian Schmitz, Beate M. Herbert, Brunna Tuschen-Caffier

**Affiliations:** 1 Department for Clinical Psychology, Psychotherapy, and Heath Psychology, University of Salzburg, Salzburg, Austria; 2 Department for Clinical Psychology and Psychotherapy, University of Freiburg, Freiburg im Breisgau, Germany; 3 Department of Health Psychology, Institute of Psychology and Education, University of Ulm, Ulm, Germany; University of Bologna, Italy

## Abstract

Many individuals restrict their food intake to prevent weight gain. This restriction has both homeostatic and hedonic effects but their relative contribution is currently unclear. To isolate hedonic effects of food restriction, we exposed regular chocolate eaters to one week of chocolate deprivation but otherwise regular eating. Before and after this hedonic deprivation, participants viewed images of chocolate and images of high-calorie but non-chocolate containing foods, while experiential, behavioral and eyeblink startle responses were measured. Compared to satiety, hedonic deprivation triggered increased chocolate wanting, liking, and chocolate consumption but also feelings of frustration and startle potentiation during the intertrial intervals. Deprivation was further characterized by startle inhibition during both chocolate and food images relative to the intertrial intervals. Individuals who responded with frustration to the manipulation and those who scored high on a questionnaire of impulsivity showed more relative startle inhibition. The results reveal the profound effects of hedonic deprivation on experiential, behavioral and attentional/appetitive response systems and underscore the role of individual differences and state variables for startle modulation. Implications for dieting research and practice as well as for eating and weight disorders are discussed.

## Introduction

In today’s industrialized societies, characterized by the omnipresence of high-energy food and a sedentary lifestyle, many individuals struggle with overweight and obesity. Also normal weight individuals are concerned about their body shape and weight, due to an unrealistically thin body shape ideal particularly in young women [1], [2]. Both cases often lead to the attempt to control or reduce weight via restriction of caloric intake. However, what seems like a straightforward act of self-regulation often turns into a boomerang: dieting might reduce weight short term but also results in a number of physiological and psychological changes that increase the probability that weight is regained in the long run, e.g., [3]. While much research effort is invested into the *physiological/homeostatic* systems underlying appetite and ingestion, the *psychological*/*hedonic* mechanisms have only recently been recognized by a wider literature, e.g., [4], [5].

### Food Deprivation: Homeostatic vs. Hedonic Processes

Biomedical research has now gathered considerable knowledge about the homeostatic effects of caloric restriction: abstinence from *any* food intake for anywhere between 2 and 48 hrs, goes along with substantial increases in appetitive responding across multiple response systems. Partially redundant gut hormone systems sense homeostatic deprivation effects and communicate with the hypothalamus and higher brain systems through several bidirectional pathways [6]. These hormonal adaptations are paralleled by changes in other responses systems such as in implicit food evaluation [7], [8], salivation [9], interoception and autonomic responding [10], [11], visual attentional processing [12], as well as neural reward system activity [13], [14], [15], [16] among others. Thus, the body attentional, experiential and motivational systems are attuned for food search to secure caloric balance.

But are all these dieting effects mediated by physiological/homeostatic systems? If hedonic factors play a role, what is their contribution to the above described deprivation effects? More broadly, is it the ‘mind or the metabolism’ (Berthoud, [17]) that drives these effects? This is difficult to answer, because homeostatic and hedonic aspects of food processing are confounded in conventional studies of food deprivation or of interindividual differences related to food intake. In the present research we introduce a manipulation of hedonic hunger through a *hedonic deprivation*, i.e. a selective restriction of a single craved food class - on the background of an otherwise unchanged food intake and therefore constant homeostatic state – and test the hypothesis that this would have substantial effects on experience, motivational/attentional physiological responding, and eating. If so, this would demonstrate the significance of hedonic factors for dieting. We chose to study chocolate deprivation because chocolate is the most commonly craved food in Western cultures (Hetherington & Macdiarmid, 1993). Furthermore, chocolate is not necessary for a nutritionally balanced diet and its consumption on top of a normal diet can therefore be attributed to hedonic reasons.

### Evidence for Hedonic Hunger

What is the evidence regarding hedonic deprivation effects? Interestingly, there is only little research that convincingly and selectively manipulated hedonic hunger. As an exception, Pelchat and coworkers have shown that young participants who were restricted to a monotonous but nutritionally adequate diet (a vanilla flavored, liquid diet) for five days experienced increased cravings for a range palatable foods [18]. A later study [19] repeated this design with a shorter monotony phase (1.5 days) and involved functional magnetic resonance imaging (fMRI) while participants imagined their favorite foods. Participants after a monotonous diet activated hippocampus, insula, and caudate more than participants on a normal diet, indicating that hedonic hunger activates brain regions implicated in sensory integration and memory. Other evidence for hedonic hunger effects is rather indirect: animal research has demonstrated that rats will work hard and endure adverse conditions to gain access to palatable food, even if standard lab chow is available [20] and that gastric filling in the absence of oral sensoric stimulation does not terminate eating [21] indicating the dominance of hedonics over homeostasis in some cases. In humans, short term experimentally induced chocolate cravings are associated with biased visual attention toward those foods [22], [23] similar to what is seen in homeostatic deprivation [24]. Last, patients with eating disorders ‘blacklist’ palatable, calorie-rich food types – even when not currently dieting. Binge eating episodes then often start with the consumption of these foods [25] suggesting that the selective deprivation increased their hedonic value.

### Interindividual Difference Variables in Food Intake

In addition to *state* effects caused by hedonic deprivation, several *traits* are likely to be influential. *Restraint eating*, for example, as measured by the restraint scale [26] indexes a pattern of chronic dieting interspersed by repletion and resultant weight fluctuations and is central to non-homeostatic eating research and subclinical eating psychopathology. A hallmark finding of restrained eating research has been that individuals high on the restraint scale, when consuming a high-calorie snack (“preload”) show increased food intake at a subsequent test meal, whereas the homeostatic response would be to decrease intake [26].

But also personality traits have been linked with eating. General *impulsivity,* for example, as measured by the Barratt Impulsivity Scale [27], is associated with test meal eating [28] and interacts with food craving in predicting inhibition deficits in a go-nogo task [29]. Impulsivity is also elevated in patients with bulimia nervosa, BN [30], binge eating disorder (BED), and obesity in adults and adolescents, see recent reviews in [28], [31], [32] and disorders of addiction [33]. A recent review suggests that particularly the subscale attentional impulsivity is associated with overeating [34] making it an interesting trait in the present context. Last, subclinical *eating disorders symptoms* might play a role in hedonic eating.

### Eyeblink Startle, Food Deprivation and Frustrative Nonreward

One measure that has gained prominence in basic emotion research and that is now increasingly applied to the food context is the eyeblink startle measure. The startle response is a translational measure used both in human and animal research, comprising a skeletomotor response to rapid onset, intense acoustic, visual, or tactile stimuli [35]. In humans, startle is measured by the strength and speed of eyelid closure to a trigger stimulus. Triggered during the presentation of highly arousing normative negative and positive images of the international affective pictures system (IAPS), it reliably differentiates aversive/defensive and positive/appetitive states [36]. Whereas early stages of picture processing reflect primarily sensory and attentional processes [37] later stages are thought to largely reflect defensive or appetitive motivation [38]. Modulation of the startle reflex involves the central amygdala [39], [40], making startle an interesting peripheral marker of this structure that is modulated by homeostatic deprivation [13], [41] [14], [42] and is heavily connected with other structures implicated in appetitive processing and craving such as the orbitofrontal cortex and the ventral striatum, reviews in [5], [43].

Several studies have applied the eyeblink startle methodology to the food and deprivation context. As one of the first studies, Drobes et al. [44] found that food pictures generally inhibit startle even in comparison to positive IAPS images, suggesting very pleasantly valenced, appetitive response. Food deprivation, however, rather than further attenuating startle resulted in an *increase* of startle, relative to satiety, indicative of a defensive, negatively valenced response. Similarly, in their second study in that paper, individuals with bulimic symptoms showed increased startle to food pictures relative to positive IAPS pictures, suggesting negative affective states, possibly due to their preoccupation with – or lack of control over - such foods. Hawk et al. [45], by contrast, found inhibited startle after 12 h food deprivation in trait food cravers (no deprivation effect in non-cravers) during presentation of in-vivo foods. With reference to the previous findings by Drobes et al., (2001), the authors speculated that real food that is directly available for eating triggers an appetitive response (in deprived trait cravers) whereas mere food picture viewing without the option of consumption triggers a state of frustrative non-reward [46] which might potentiate eyeblink startle. Rejeski et al. [47], studying startle in non-deprived individuals, confirmed that food cue exposure can trigger negative affect in individuals experiencing state craving, especially when they expect a long delay until consumption. Studies in eating disordered individuals revealed that additional factors modulated startle, such as disorder subgroups (anorexia or bulimia nervosa, [48]) and acute homeostatic deprivation/satiation [49]. In sum, research has documented the utility of the eyeblink startle measure in the context of food image processing but highlighted that several state (craving, deprivation, frustration) and trait (symptoms of disordered eating) moderators exert influence and make its interpretation more complex.

### The Present Study

The present study explored the state of hedonic deprivation by asking regular chocolate eaters to refrain from consuming chocolate for one week. In two experimental sessions before and after this deprivation, we measured eyeblink startle to *chocolate* images in addition to experiential and behavioral measures. Non-chocolate but high-energy, savory *food* pictures served as control images to test for the specificity of deprivations effects. A test meal assessed actual consumption of the types of chocolates and foods represented by these images to assess the behavioral effects of the manipulation. Eyeblink startle was also assessed during the intertrial intervals which served as reference category. We further measured several potential state (strength of experienced deprivation, frustration/depression) and trait moderators (impulsivity, eating disorder symptoms, restraint eating) of startle responding.

We expected increased ratings of palatability and desire to eat for chocolate images as well as increased chocolate consumption during hedonic deprivation compared to satiation, based on research on homeostatic/hedonic deprivation effects introduced above. Predictions for startle responses during chocolate images were more difficult, common sense would suggest appetitive startle attenuation during hedonic deprivation but the literature on homeostatic deprivation would predict either startle potentiation (Drobes et al., [44]) or attenuation (Hawk et al., [45]), depending on whether frustrative states arise. Thus, we stated no directional hypothesis for startle but expected an association of startle with the subjective response to the manipulation, particularly craving [47], experience of frustration, and interindividual differences/eating disorder symptoms [44], [48], [49].

## Methods

### Participants

Participants were 29 females who reported no current mental disorders on a telephone screening interview based on the structure clinical interview for DSM-IV disorders [50]. Further exclusion criteria were lifetime eating disorder, bipolar, psychotic, or borderline personality disorder, diabetes, current diet (which would preclude satiety) or chronic medication use. It was further required that participants liked chocolate at least with an intensity of 80 on a 0–100% scale and ate chocolate at least three times per week on average.

### Questionnaires

The *Eating Disorder Examination Questionnaire, EDE-Q;* [51] is a 36-item self-report measure that assesses the severity of eating pathology with four subscales (restraint eating, eating concern, weight concern and shape concern) and a total score. The total score and the subscales show high internal consistency, stability, and validity in the English and German version [51], [52] and excellent internal consistency in the present sample (Cronbach’s alpha = .925).

The *Restraint Scale, RS;* [26] is a 10-item measure with scores ranging from 0 to 35. It probes for concerns for dieting and weight fluctuations. The German version of the RS has good psychometric properties, Cronbach’s alpha = .83, [53] which was confirmed in the present sample (Cronbach’s alpha = .855).

The *Barratt Impulsiveness Scale, Version 11 (BIS-11)* assesses impulsiveness on 30 items. Several studies confirmed the acceptable psychometric properties of the BIS-11 in the English [27], [33] and German version Cronbach’s alpha = .69, [54], [55]. Cronbach’s alpha in the present sample was good (.826). The subscale attentional impulsivity similarly showed good internal consistency (.714). Questionnaires were administered online prior to the first laboratory session.

The German version of the Spielberger State-Trait Anxiety Inventory, state version, STAI-S; [56] was administered at the beginning of each session to test for anxiety as a possible confound of deprivation effects.

### Ethics Statement

The study was approved by the ethics committee of the University clinic of Freiburg and written informed consent was obtained from all participants. Before participation, experimental procedures were described in detail. Participants were informed that they could stop the experiment at any time with full compensation.

### General Procedure

All participants completed an informed consent form that had been approved by the local ethics committee and received €50 for their participation. In a within-subject design, participants underwent testing in a *chocolate-deprived state* and a *chocolate-satiated state* (state order counterbalanced across participants). Instructions (given at the lab or during screening depending on order) for the satiated phase (7 days +−1 day) required participants to continue eating chocolate as they would normally do, whereas instructions for the deprived phase (7 day +−1 day) asked participants to refrain from eating chocolate or anything containing chocolate. Participants completed a detailed daily diary during both the deprivation and satiation periods and this diary was reviewed with the participant at the beginning of each lab session. Sessions were scheduled between 2 pm and 4 pm to limit circadian variations. Overall deprivation compliance was very good: 86.2% of participants showed 100% compliance, the remaining ate chocolate between 1 and 2 times in during the deprivation period. Likewise, during satiety, 82.7% of participants ate chocolate > = 5 times/week, 10.3% 4 times and 7.00% 3 times per week. Lab session were separated by 7 days (M = 7.0, SD = 0.377, range 6–8).

### Picture Viewing Task

After being welcomed and shown around the laboratory, participants completed the STAI-S and were then fitted with the EMG electrodes and seated inside a sound attenuated chamber to complete an adaptation phase (4 min) and a go-nogo task (∼20 min, results not shown). A startle habituation phase (5 probes, 11.5 s +−1.5 s intertrial interval, ITI) preceded the picture viewing task. Two *food-types* were presented in two blocks of 20 images (as one exploratory experimental factor was perceived food availability, the experimenter indicated that the foods in one of the blocks would be available for consumption after the task)*:* Each block comprised a different set of 10 pictures of high calorie but non-chocolate food pictures (waffles, nuts, pretzels, non-chocolate candy, popcorn, potato chips etc., see details in *supporting information about image selection* [57]) and 10 chocolate pictures (e.g., chocolate-containing cakes, donuts, chocolate bars) in individually randomized order, resulting in a total of 40 picture trials. Pictures were presented for 6 s and were separated by a 10–12 s ITI. During 70% of the picture trials, startle probes were presented, either at 3.5 s (43%) or 5.5 s (57%) after picture onset. Furthermore, startle probes were presented during 20% of the ITIs (8 startles, at 6 s). After each block, participants used visual analogue scales to rate whether they felt “frustrated” or “depressed” (“0- not frustrated/depressed at all” to “10- very frustrated/depressed”) during the previous block. During a subsequent rating phase, each picture was shown again and rated on visual analogue scales on palatability (“0- not palatable at all” to “10- very palatable,”) and desire to eat (“0- don’t like to eat this now” - “10- would like to eat this now”).

### Taste Test

After the task a subset of food items, corresponding with the chocolate and food pictures (with equal numbers/quantities of food and chocolate items) was presented. Participants were asked to taste and rate the foods and chocolates on palatability. Subsequently participants were encouraged to ‘help themselves’ with the food while the experimenter left the room. Unbeknownst to the participants, remaining food items were later weighted to calculate the amount eaten of each food-type (food, chocolate). Participants were subsequently debriefed about this procedure.

### Apparatus and Startle Analysis

Startle tones were 50 ms instantaneous onset white noise sound bursts delivered via insert earphones calibrated to 103 db. Two miniature EMG electrodes were placed on the orbicularis oculi of the right eye following established conventions [58]. EMG was recorded at 500 hz using SynAmps amplifiers and Scan 4.0 software (Neuro-Scan, Inc., Sterling, Virginia, USA). Offline preprocessing was done in ANSLAB software [59] and comprised high-pass filtering (28 Hz), notch filtering (50 Hz), rectification, and low pass filtering (16 Hz) of the EMG signal. Startle response magnitude (peak EMG response in microVolts) was calculated as the difference between the peak EMG response within 20 to150 ms after probe onset and startle baseline, scored as the mean EMG in the 50 ms window before startle onset. Trials with no observable startle response were scored as zero magnitude unless an unstable baseline prohibited startle detection - scored as missing value. Valid, non-zero startles were detected on 91.4% of the probes.

### Statistical Analyses

*Consumed calories* during the post-experimental taste test, and subjective *palatability* and *desire to eat* ratings were separately subjected to 2×2, State (satiated, deprived) × Food-type (chocolate, food) repeated measures analysis of variance (ANOVA). Block level ratings of frustration and depression were averaged into a *frustration/depression* score (Cronbach’s alpha = .931) and submitted to paired sample *t*-test between states. Preliminary analyses of *startle magnitude* during ITIs revealed higher values during deprivation compared to satiation (M_DEP_ = 33.2 mV, SD = 31.0, M_SAT_ = 19.7, SD = 12.5, *t*(26) = 2.48, *p* = .020, *d* = 0.57), thus each session’s ITI startles were subtracted from respective image startles to corrected for these background state effects. These differential image startles were then submitted to a 2×2, Food-type (chocolate, food) × State (satiated, deprived) ANOVA. There were no significant order effects (first deprived, first satiated) on any of the measures. Generally, orthogonal *t*-tests between states (deprived vs. satiated) were used to follow up on interactions. We report partial eta square (*η_p_* ^2^) for significant ANOVA results, and Cohens’ *d* for *t*-tests. Alpha levels for exploratory correlational analyses were Bonferroni corrected.

## Results

### Manipulation Check

#### Consumed calories

**Figure 1:**
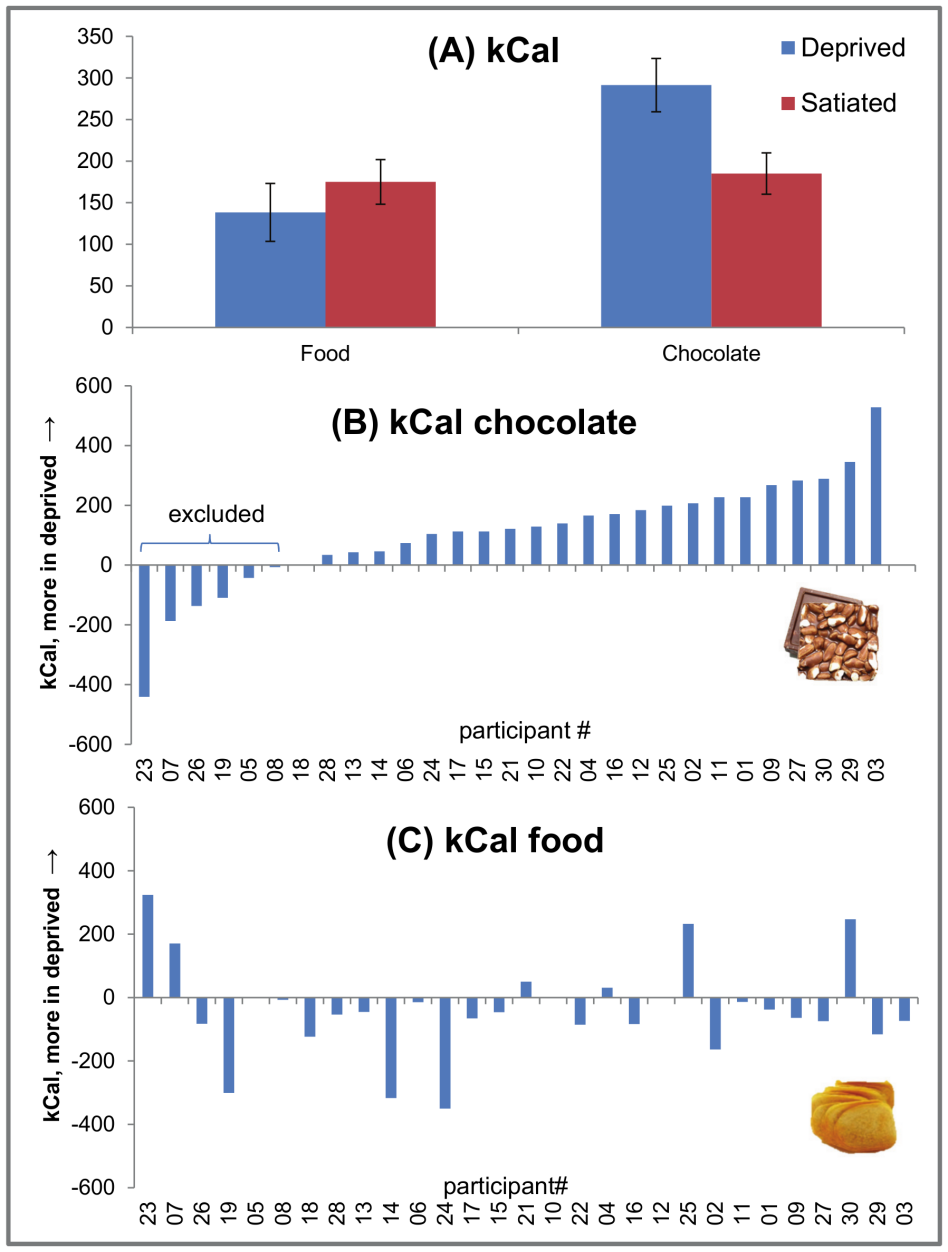
Number of calories consumed during manipulation check as a function of state (deprived, satiated) and food type (chocolate, food) (A). Six participants showed opposite effects of consuming more chocolate during satiation and were therefore excluded (B). No systematic deprivation effects were seen for consumption of non-chocolate foods in excluded or included participants (C).

The State×Food-type ANOVA on calories consumed after the experimental sessions revealed a trend toward a main effect of State, *F*(1,28) = 3.07, *p = *.090, *η_p_* ^2^ = .099, and a significant main effect of Food-type, *F*(1,28) = 5.47, *p = *.027, *η_p_* ^2^ = .1640, which interacted with State, *F*(1,28) = 8.90, *p<*.006, *η_p_* ^2^ = .241. Post-hoc *t*-tests revealed that during deprived state, as expected, participants consumed more chocolate than during satiated state, *t*(28) = 3.12, *p* = .004, *d* = 0.68. No such effect was found for non-chocolate foods, *t*(28) = 1.31, *p* = .199 (see Figure 1A). Unexpectedly, as illustrated in Figure 1B, six participants responded to the manipulation in the reverse direction: they consumed more chocolate in the satiated than in the deprived state and were therefore excluded from further analyses (similar albeit slightly weaker results were obtained when including all participants). Figure 1C illustrates consumption of non-chocolate foods across participants - no (compensatory) relationship with chocolate consumption was evident in included or excluded individuals.

### Psychometric Characteristics

The 23 remaining participants (8 psychology students, 15 students in other degrees) had a mean age of 23.0 (SD = 4.66, range 18–39) and normal average weight (mean Body Mass Index [BMI] = 21.8, SD = 3.78, range = 17.5 to 35.2, exclusion of two overweight (28.1, 27.0) and one obese participant (35.2) did not influence the results) as well as a wide range on RS scores (M = 11.0, SD = 5.62, range 2–20), with 5 participants being considered restrained eaters (scores < = 16, [53]). Similarly, we noted significant variability on the EDE-Q, (M = 1.84, SD = 0.71, range 1–3.87), but importantly, none of our participants scored above the cutoff sometimes used for clinical eating disorders of < = 4 [60], [61]. A relatively wide range on the BIS-11 was notable (M = 58.9, SD = 9.56, range 50–82): according to Stanford et al., [33], normal healthy participants range 52–71, thus 2 of our participants can be considered impulsive (no cutoff data available for the attentional impulsivity subscale). STAI-S scores were similar during deprived (M = 36.4, SD = 6.73) and satiated (M = 35.3, SD = 7.14) states, supporting comparable anxiety related affect at both sessions, *t*<1.00.

### Food Image Ratings: Desire to Eat and Palatability

**Figure 2:**
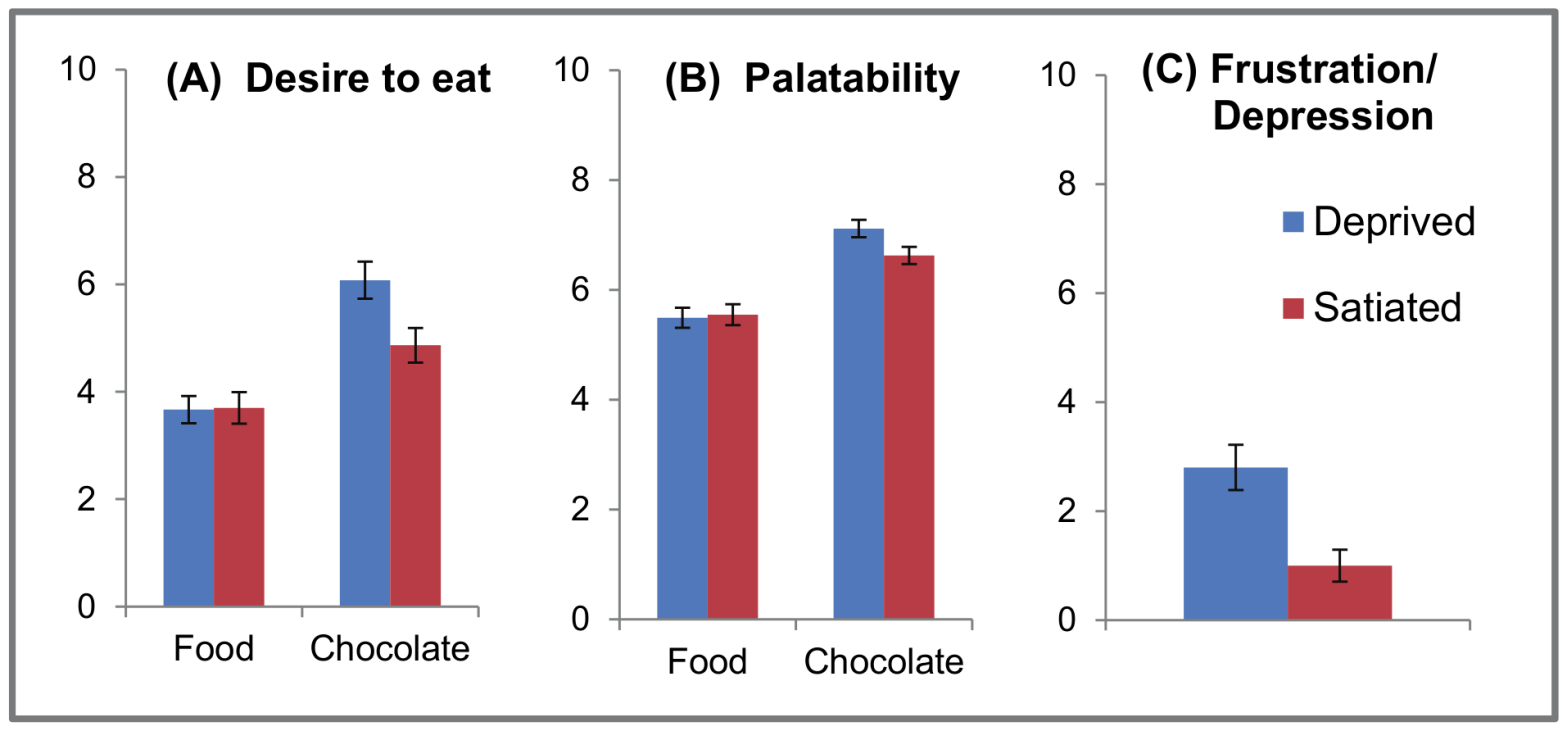
Subjective ratings as a function of state (deprived, satiated) and food type (chocolate, food): Picture-wise desire to eat (A), and palatability (B) ratings and block-wise ratings of frustration/depression (C).

The State×Food-type ANOVA on *desire to eat* ratings yielded significant main effects of State, *F*(1,22) = 7.56, *p = *.012, *η_p_* ^2^ = .256, Food-type, *F*(1,22) = 45.3, *p<*.001, *η_p_* ^2^ = .673, and a State×Food-type interaction, *F*(1,22) = 10.0, *p = *.004, *η_p_* ^2^ = .313. Participants reported a stronger desire to eat the presented chocolate items during deprivation, compared to satiation, *t*(22) = 3.48, *p* = .002, *d* = 0.73, while no such effect was found for food, *t*<1.00 (see Figure 2A).

A very similar pattern was obtained for *palatability*: the State×Food-type ANOVA yielded significant main effects of State, *F*(1, 22) = 4.92, *p* = .037, *η_p_* ^2^ = .183, and Food-type, *F*(1,22) = 34.3, *p<*.001, *η_p_* ^2^ = .611, and a significant interaction, *F*(1,22) = 11.2, *p = *.003, *η_p_* ^2^ = .338. Participants rated palatability of chocolate as higher when deprived, *t*(22) = 3.33, *p* = .003, *d* = 0.67. No such effect was found for food, *t*<1.00 (see Figure 1B).

### Ratings of Frustration/Depression

Ratings of frustration/depression were elevated during deprivation relative to satiation, *t*(22) = 3.166, *p* = .004, *d* = 0.70 (Figure 2C).

### Eyeblink Startle Data

**Figure 3:**
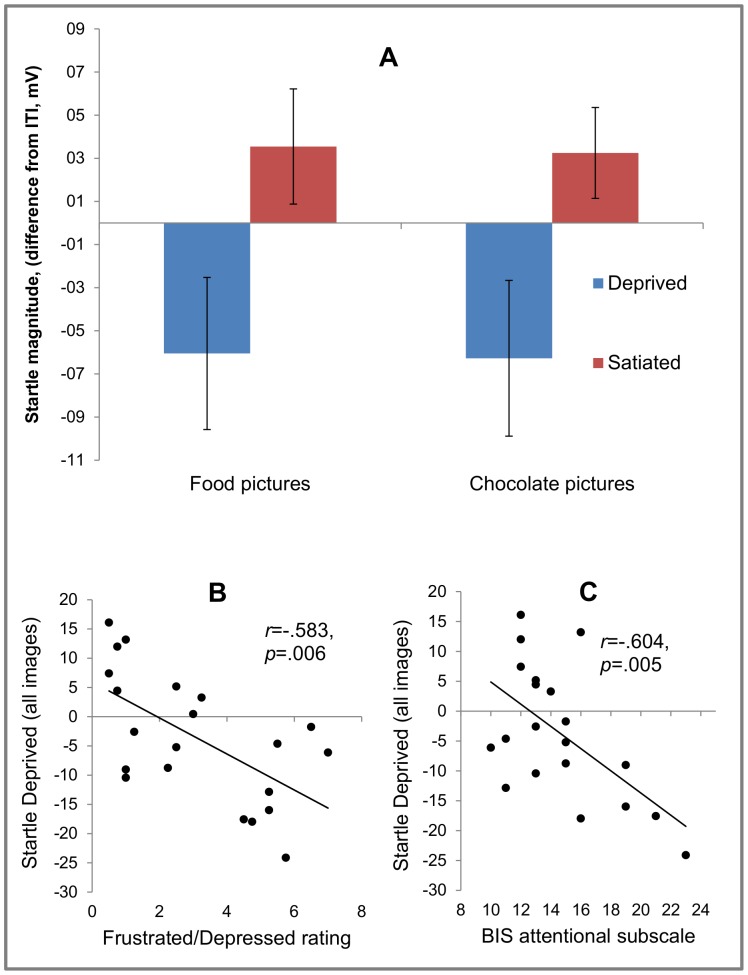
Eyeblink startle magnitude (image – ITI) as a function of state (deprived, satiated) and food type (chocolate, food) (A). Startle magnitued, collapsed across food type, correlated negatively with state measures of frustration/depression (B), and attentional impulsiveness (BIS, Barrat Impulsiveness Scale) (C).

The State×Food-type ANOVA yielded a main effect of State, *F*(1, 20) = 5.34, *p* = .032. *η_p_* ^2^ = 0.21 but no State×Food-type interaction, *F<1.00* Startle magnitude was attenuated during deprivation compared to satiation regardless of food type (Figure 3A). Correlational analyses were used to characterize the deprived state using startle data averaged across food and chocolate images.

### Correlational Analysis

Two sets of correlational analyses were conducted to characterize image startle responding in the deprived state with regard to self reported state and trait measures. Regarding *state* measures we had hypothesized that frustration/depression and palatability/desire to eat ratings would be associated with startle responding. In fact, participants with attenuated startle responding during deprivation also reported elevated frustration/depressing during picture viewing, *r*(21) = -.583, *p* = .006 (Figure 3B). Ratings of palatability and desire to eat were uncorrelated with startle (alpha level set to 0.05/3 = .0167).

A second set of correlations investigated individual differences in more trait-like measures of eating psychopathology (EDE-Q, RS) and impulsivity (BIS-11) with startle responses during deprivation. The RS and EDQ-Q scales were unrelated to startle responses, however higher scores on the attentional impulsivity scale of the BIS-11 scores were associated with attenuated startle responses, *r*(20) = −.604, *p* = .005 (alpha = .0167, Figure 3C, one missing value on BIS-11, correlation total BIS score: *r*(20) = −.49, *p* = .028).

## Discussion

Dealing with cravings for high-energy snacks such as chocolate represents a tremendous challenge in today’s societies characterized by omnipresence and low-cost availability of these foods, a sedentary lifestyle and an unrealistically thin body image ideal. A common response to this environment is dieting or a selective reduction of high-calorie foods, such as chocolate. Thus, the relevance of chocolate cravings for eating disorders, overweight and obesity is evident. To our knowledge ours is the first study using physiological measures to evaluate effects of a selective chocolate deprivation, a procedure which we have termed *hedonic deprivation*. The results can be summarized as follows.

The behavioral effects of one week of chocolate-‘abstinence’ – in the presence of otherwise unchanged diet – were clearly evident: participants liked (palatability), wanted (desire to eat), and consumed chocolate more than other high-energy savory foods when hedonically deprived compared to when satiated. Importantly, deprivation modulated the eyeblink startle response: intertrial interval startle increased during deprivation compared to satiation. Image startles – expressed as difference to this intertrial interval - showed a relative reduction during images, reflecting a more appetitive/approach-related response and/or more attention allocation to these cues during deprivation relative to satiation. This effect was not selective, however: both chocolate images and closely matched, palatable high-energy non-chocolate food images resulted in a similar relative reduction of startle response. Correlational analyses of ITI-picture difference scores demonstrated that stronger relative startle attenuation went along with frustration/depression during deprivation. Stronger relative startle attenuation was also associated with higher scores on attentional impulsivity (BIS subscale).

### The Simple Picture: Hedonic Deprivation Drives Appetitive Responses

The behavioral and subjective effects of the hedonic deprivation are generally consistent with findings that a monotonous diet increases cravings for a range of palatable foods (Pelchat et al., 2000). In contrast to this previous study our diet manipulation was rather subtle and selective: only chocolate needed to be avoided without any restrictions on other sweet, tasty or otherwise palatable foods. This hedonic deprivation of one food class was sufficient to affect experience and behavior in the same direction as homeostatic hunger. This means that the homeostatic, afferent signaling of energy repletion is not a necessary prerequisite for the experience of wanting, liking, and eating in line with the hedonic hunger literature reviewed above. That said, we also noted some variability, six of our participants ate less chocolate when deprived. In fact not all previous studies have found a relationship with dietary restraint and craving [62], [63] [64] and the monotonous diet in Pelchat et al. (2000) increased craving in most but not all participants.

In those participants responding with increased chocolate consumption to the hedonic deprivation startle magnitudes were reduced during chocolate images relative to ITI. This is in line with an appetitive response, as found, e.g. by Hawk et al., (2004) in 12 h food deprived individuals. However, startle attenuation was also seen for non-chocolate food pictures indicating a ‘spill over’ or generalization effect. This might suggest that deprivation of one particular food class has wider effects on the more implicit levels represented by startle responses here as on subjective and behavioral levels. One possible explanation for this observation is that the hedonic deprivation manipulation increased the participants’ self-monitoring of general food and snack intake, since they had to make repeated ‘allowed - not allowed’ decisions. This attentional focus to, and increased salience of, the whole category of palatable energy dense foods might have driven the generalized startle attenuation. The fact that startle is modulated by the amygdala, now largely understood as salience/biological relevance indicator [65] also points to potential *attentional interpretations* of attenuated startle [37], [38]. Thus, the amygdala might compute enhanced salience of the a whole class of energy rich foods but other parts of the part of the brain’s reward system might chime in to drive the chocolate specific behavioral and experiential responses observed here. An alternatively explanation for the generalized startle response is methodological: participants might have also restricted consumption of the palatable and energy dense items contained in the ‘food’ category, even though they were not instructed to do so. Last, food restriction can increase thoughts about ‘forbidden fruits’ through ironic processes: what needs to be suppressed becomes more available [66], [67], [68]. These ironic processes could have made the whole category of high-calorie, palatable snacks more available or attractive. In any case, future research would profit from including a non-food control condition to gauge reliability and breadth of such generalization of startle attenuation.

### The more Complex Picture: Differential Startle Responses and Negative Experience

However, hedonic deprivation did also have an aversive side: frustration/depression ratings were higher during deprivation as were ITI startle responses. Furthermore, more ‘frustrated’ and ‘impulsive’ participants showed a stronger image startle attenuation relative to their ITI startles. Yet, can the state of deprivation be appetitive and aversive at the same time? In fact, this observation dovetails with the previous startle literature, suggesting that deprivation can create defensive and frustrated states (Drobes et al., 2001) and is subject to individual differences in response to the food restriction (Hawk et al, 2004, Rejeski et al. 2010). In fact a host of studies has linked appetitive responses like overeating with negative moods [69], [70] and food cravings have been described as being characterized by co-activated incompatible mood states, such as approach inclinations (favoring consumption) and avoidance inclinations (favoring restraint) [71]. For example, chocolate cravers react more joyfully to chocolate exposure when deprived but also more guiltily [72] and non-deprived chocolate cravers show startle potentiation but a non-defensive cardiac response pattern to chocolate images [10], revealing a differentiated physiological response. Last, it appears plausible that the symbolic nature of the images shown here also drives frustrative background states when actual consumption is delayed [46].

The issue of appetitive responding and craving on the one hand, and concurrent frustrative/defensive states on the other during deprivation dovetails with the ongoing discussion on the issue of food addiction [73], [74]. It is known from other addictions that rewarding aspects of the substance dominate early addiction stages, but negative background states emerge during later ‘withdrawal’ stages, punctuated by acute, appetitive substance anticipation. This sequence is seen by some researchers to be present in food addiction as well [75] making the question of whether hedonic deprivation can give rise to addictive tendencies a worthwhile future direction.

### Impulsive Individuals are ‘at Risk’ during Hedonic Deprivation

Relative startle inhibition was stronger in individuals with higher scores on attentional impulsivity, capturing a general inability to focus attention or concentrate. This subscale has shown the most reliable association with overeating [34], possibly because affected individuals are susceptible to attentional capture by palatable foods [76]. General impulsivity measured by the BIS scale has been linked with eating disorders, in particular BN [30], [77], BED and obesity in adults and adolescents, see recent reviews in [31], [32], but also with substance use, addictive disorders and other disorders [33]. Furthermore, in healthy individuals the BIS-11 scale is associated with the disinhibition scale of the three factor eating inventory [78], taste-based food choices [79], and various experimental tasks assessing behavioral inhibition and delay discounting [80]. BIS-11 scores interact with perceived availability and response inhibition in predicting cue elicited craving in alcohol dependence [81], [82], [83]On this background, the present data suggest that attentional impulsivity might threaten diet-adherence and warrant special attention for these individuals.

### Limitations and Future Directions

Our study has some obvious limitations which should be addressed in future research. First, only young, educated women were studied here. We had selected this population based on their elevated risk for restrained eating and eating disorders but these factors did not prove influential in this study. Thus, extension toward a mix-sex sample with a broader age range and representative education levels is desirable. Second, although behavioral (consumed calories), and rating (palatability, desire to eat, depression/frustration) data showed clear deprivation effects in the full sample, we decided to restrict our detailed analysis to those participants who actually consumed more chocolate after deprivation (excluding six participants). Thus, future research on hedonic deprivation needs to take some individual differences in response to the hedonic deprivation into account. Third, the possibility exists that the strong state effects on ITI-startle responses resulted in ceiling/floor effects in some individuals with the result that their image startles regressed toward the mean. Although the meaningful and strong correlations of the picture - ITI startle difference scores (with impulsivity and frustration/depression) would argue against this, the possibility remains that comparable ‘baselines’ (ITI startles) could result in a different response pattern during picture viewing (i.e. startle potentiation during deprivation as in Drobes et al. [44]). Future research should extend baseline startle assessment and also match pictures and ITI presentations with regard to probe frequency (which was unbalanced here). In addition, startle should be complemented by biological measures from other domains (e.g. neurocognitive, autonomic, neuro-endocrine, metabolic) which might help in understanding the ambivalent, multifaceted state of deprivation and its hedonic and homeostatic constituents. Complementing startle with event related potentials, for example, would help in estimating the contribution of attentional processes (measured, e.g. though the P3 amplitude to image and startle probe [38]) to startle modulation. Fourth, since the present project tested several related questions, participants had completed a go-nogo task prior to the present task. It is possible that this involved certain priming effects or reduced concentration. However, these would affect both sessions to a similar degree. Last, conceptually, this research was inspired by the observation that eating disordered patients hedonically deprive themselves through ‘blacklisting’ chocolate and other high-energy foods with the result that eventual consumption of these foods often trigger binge eating (loss of control). Successful cognitive behavioral treatment involves a reintegration of such foods into the diet to reduce such deprivation effects. Thus eyeblink startle might be useful as an outcome measure of such treatments for eating disorders.

### Conclusions

The profound effects of one week of voluntary chocolate deprivation on experience, behavior and startle responding highlights the power of hedonic determinants of eating and mandates caution with regard to banning craved high-calorie foods during dieting [84]. The response to such selective dieting renders these foods more salient and/or appetitive while at the same time inducing aversive experiences and defensive background states. Impulsive individuals, and those responding with strong frustration/depression to deprivation are at elevated risk and might ultimately break their diet if not receiving extra guidance in prevention and treatment, as for example through one of the recently suggested treatments of impulsive eating behavior [85]. Hedonic deprivation needs future study to inspire possible alternative ways to treat overeating, bingeing and obesity.

## Supporting Information

Footnote S1 **Image selection.**
(DOCX)
