# Supplementary material for: Startling Sweet Temptations: Hedonic Chocolate Deprivation Modulates Experience, Eating Behavior, and Eyeblink Startle
Source: PLoS One. 2014 Jan 9;9(1):e85679. doi: 10.1371/journal.pone.0085679 (PMC3887070; doi:10.1371/journal.pone.0085679)
Supplement: Footnote S1 — Image selection. (DOCX) [file pone.0085679.s001.docx]

*Footnote S1: Image selection*

Pictures were taken from the food.pics database (Meule & Blechert, 2012, freely available on www. food-pics.sbg.ac.at), numbers of Chocolate images were 4, 158, 159, 160, 161, 162, 163, 164, 165, 166 (block A) and 41, 137, 167, 168, 169, 170, 171, 172, 173, 174 (block B). Food image numbers were 9, 63, 109, 110, 124, 146, 147, 148, 149, 150, (block A) and 26, 117, 21, 151, 152, 153, 154, 155, 156, 157 (block B). Displayed food and chocolate did not differ on Kcalories, (161 vs. 229, t(38)=1.72, p=.085) or carbohydrates (in grams 23.7±19.3 vs. 26.9±16.3, t>1.00) but chocolate contained more fat than food (in grams 6.30±6.07 vs. 12.1±9.45, t(38)=2.28, p=.028).
